# Lipoprotein(a) in Cardiovascular Diseases: Insight From a Bibliometric Study

**DOI:** 10.3389/fpubh.2022.923797

**Published:** 2022-07-05

**Authors:** David Šuran, Helena Blažun Vošner, Jernej Završnik, Peter Kokol, Andreja Sinkovič, Vojko Kanič, Marko Kokol, Franjo Naji, Tadej Završnik

**Affiliations:** ^1^Department of Cardiology and Angiology, University Medical Centre Maribor, Maribor, Slovenia; ^2^Faculty of Medicine, University of Maribor, Maribor, Slovenia; ^3^Community Healthcare Centre Dr. Adolf Drolc Maribor, Maribor, Slovenia; ^4^Faculty of Health and Social Sciences Slovenj Gradec, Slovenj Gradec, Slovenia; ^5^Alma Mater Europaea, Maribor, Slovenia; ^6^Faculty of Electrical Engineering and Computer Science, University of Maribor, Maribor, Slovenia; ^7^Department of Medical Intensive Care, University Medical Centre Maribor, Maribor, Slovenia; ^8^Semantika Research, Semantika d.o.o., Maribor, Slovenia

**Keywords:** lipoprotein(a), bibliometrics, synthetic knowledge synthesis, atherosclerosis, cardiovascular disease, inflammation, post-menopausal women, calcific aortic valve stenosis

## Abstract

Lipoprotein(a) [Lp(a)] is a complex polymorphic lipoprotein comprised of a low-density lipoprotein particle with one molecule of apolipoprotein B100 and an additional apolipoprotein(a) connected through a disulfide bond. The serum concentration is mostly genetically determined and only modestly influenced by diet and other lifestyle modifications. In recent years it has garnered increasing attention due to its causal role in pre-mature atherosclerotic cardiovascular disease and calcific aortic valve stenosis, while novel effective therapeutic options are emerging [apolipoprotein(a) antisense oligonucleotides and ribonucleic acid interference therapy]. Bibliometric descriptive analysis and mapping of the research literature were made using Scopus built-in services. We focused on the distribution of documents, literature production dynamics, most prolific source titles, institutions, and countries. Additionally, we identified historical and influential papers using Reference Publication Year Spectrography (RPYS) and the CRExplorer software. An analysis of author keywords showed that Lp(a) was most intensively studied regarding inflammation, atherosclerosis, cardiovascular risk assessment, treatment options, and hormonal changes in post-menopausal women. The results provide a comprehensive view of the current Lp(a)-related literature with a specific interest in its role in calcific aortic valve stenosis and potential emerging pharmacological interventions. It will help the reader understand broader aspects of Lp(a) research and its translation into clinical practice.

## Introduction

Low-density lipoprotein (LDL) cholesterol (LDL-C) and other apolipoprotein B-containing lipoproteins have been extensively studied, and abundant evidence confirms their causal role in atherogenesis (1). The atherogenic potential of lipoprotein(a) [Lp(a)] has been increasingly recognized in the last decade. Lp(a) is a complex polymorphic lipoprotein structurally similar to the LDL-particle with an additional apolipoprotein(a) [apo(a)] connected by a disulfide bond (2–4). Its serum concentration is mostly genetically determined and only modestly influenced by diet and other lifestyle interventions. Several unique properties of Lp(a) can be attributed to the presence of apo(a) (3), yielding its pro-atherogenic, pro-inflammatory, and potentially pro-thrombotic potential (2). Extensive evidence suggests Lp(a) as a pre-disposing factor for atherosclerotic cardiovascular disease (ASCVD) (1, 2, 4). More recent studies have also revealed a link between Lp(a) concentration and calcific aortic valve stenosis (CAVS). A meta-analysis of population studies demonstrated a significantly increased ASCVD risk in patients with Lp(a) concentrations above 30 mg/dl (5).

Lipoprotein(a) screening recommendations are still a matter of debate. The current guidelines of the European Society of Cardiology recommend Lp(a) measurement at least once in each adult person's lifetime to identify those with very high inherited levels >180 mg/dl, which is supposed to be an ASCVD risk equivalent to heterozygous familial hypercholesterolaemia. Lp(a) measurement is crucial in patients with pre-mature ASCVD and for reclassification in patients between moderate and high ASCVD risk (6).

Despite the intensity of lipoprotein research, there is no overall and holistic understanding of Lp(a) biology (5). The benefit of Lp(a)-lowering drugs on clinical outcomes like reducing major adverse cardiovascular events and mortality has not been demonstrated yet. The National Heart, Lung, and Blood Institute identified various challenges in Lp(a) research, one of the most important being the lack of globally standardized Lp(a) assays (6).

Commonly used methods to comprehensively assess research activity in any scientific field regarding research content and spatial entities are bibliometrics and knowledge synthesis; triangulating both leads to the so-called synthetic knowledge synthesis, which enables one to analyse large corpora of scientific publications (7). Bibliometrics enables one to analyse the scientific literature production on a particular field using mathematics, bibliometric software, and statistical tools to identify (a) key research features such as literature production, trends, more prolific research entities (i.e., countries, institutions or authors), the intensity of Co-operation or spatial distribution of research; (b) content of the research literature production like research themes, hot topics, history of knowledge development or influential papers; and (c) other relevant research patterns of interest such as citation patterns (7). To review the spatial features, content and state of the art in Lp(a) research, we performed synthetic knowledge synthesis of the large corpora of Lp(a) related publications (8). Thereby, a framework for easier understanding of the Lp(a) research volume, scope and content was established, and a foundation for employing more formal (and traditional) knowledge synthesis methods was constructed.

### Aim of the Study

Our study aimed to answer the following research questions regarding Lp(a) in cardiovascular disease (CVD) research:

- Is the volume and scope of research sufficient in terms of its importance?
- Is the research between countries (focusing on developed and less-developed countries) sufficiently dispersed to support World Health Organization Global health initiatives and optimal country Co-operation and knowledge transfer?
- What are the most prolific source titles to inform one about the research and where to publish his/her research?
- What are the most prolific research themes to be translated into practice?
- Which research gaps and future research directions can be identified?

## Methodology

### Bibliometric Analysis

One of the bibliometric approach pioneers Alan Pritchard, defined bibliometrics as “the application of mathematical and statistical methods to books and other media of communication” (9). Pritchard's definition was later extended to define bibliometrics as “the quantitative analysis of the bibliographic features of a body of literature” (10). Kokol et al. (8) showed that bibliometrics had its roots already at the end of the 19th century and that it has been widely and successfully employed in medicine. While generally, bibliometrics is not used to generate new basic medical knowledge, it can produce novel meta-knowledge regarding specific medical areas, catalyzing new knowledge development and streamlining basic medical research. Examples of usages would include synthesizing knowledge of the area, assessing the maturity of research, providing guidance on feasibility of further research in specific areas of interest, identifying good or interesting themes and best information/knowledge sources, identifying with whom to cooperate or where to publish to make the research influential. Bibliometrics can be used standalone or complementary to systematic, integrative, scoping and similar reviews for quantitative analyses of research literature production.

### Knowledge Synthesis

Bibliometrics can be also used as a part of synthetic knowledge synthesis—a combination of bibliometric mapping and content analysis (11). Unlike traditional and more formal knowledge synthesis methods (such as meta-analysis, systematic or literature reviews, to name a few), which are usually performed manually, are labor intensive and are usually limited to a relatively small number of publications (typically <100 publications). Synthetic knowledge synthesis enables one to process several thousands of publications. The broadness above makes it a natural fit as the starting point for researchers to identify new possible areas of interest or areas that may have received insufficient attention in previous research.

Bibliometric mapping visualizes the research literature production and content with various bibliometric maps/landscapes (12). Bibliometric mapping is based on text mining and Co-word analysis, which captures the frequency of pairs of words or phrases in and between documents and analyses them using clustering algorithms. VOSViewer (Leiden University, Netherlands) (13, 14) is a widespread tool for bibliometric mapping. VOSviewer software combines closely associated terms into clusters denoted by the same cluster color; the term proximity can then be interpreted as a sign of their similarity. VOSviewer also allows the creation of landscapes in which terms are colored according to the average year of the term's appearance in the scientific literature. Term popularity is indicated by the size of the node in the landscape. Our study used bibliometric mapping landscapes generated by VOSViewer as an input to the thematic analysis to analyse the content of literature production regarding the role of lipoproteins in CVD. Additionally, a descriptive bibliometric analysis was performed.

### Identification of Historical Roots

Historical roots are important and influential publications in a specific research area (SRA) [in our case the Lp(a) research]. The number of citations that SRA publications received might seem a good metric to identify SRA's historical roots, but unfortunately, that is not true. Namely, SRA publications might have been cited mainly by publications outside the SRA in question, meaning that such publications are not influential in the SRA in question. Moreover, the SRA's publication citations may not be indexed by bibliographic databases. A method called References Publication Years Spectroscopy (RPYS) has been developed to overcome this problem and has already been successfully used in medicine (15). RPYS analyse references' publication years and aggregate the number of cited references over time on a spectrogram. Pronounced peaks indicate the years when historical roots/ papers were published.

### Forming the Corpus

Corpus was harvested on July 16th, 2021, from the Scopus bibliographic database using the search string [“lipoprotein(a)” or “lp(a)”] and (“cardiovascular dis^*^” or CVD) for the whole period covered by Scopus (without exclusion criteria for the year of publication). We used the Scopus built-in services to perform the descriptive bibliometric analysis (distribution of documents, literature production dynamics, and most prolific source titles, institutions, and countries). RPYS was performed with the CRExplorer software (15). The corpus in the CSV file format was exported to the VOSViewer software, used to generate the term cluster landscape and the authors' keyword cluster landscape. Thematic analysis (16) on both landscapes assigned themes to clusters. In order to identify hot topics, author keyword networks for 2016–2017 and 2018–2020 were compared as described by Kokol et al. (17).

## Results and Discussion

### Volume and Historical Scope of the Lp(a) Research in CVD

The search resulted in 3,547 publications within nine document types. Research articles were the prevailing document type within 2,296 publications. The next most frequent type of documents were reviews (*n* = 845), followed by editorials (*n* = 112), conference papers (*n* = 93), letters (*n* = 72), notes (*n* = 68), short surveys (*n* = 42), book chapters (*n* = 19), and one erratum.

**Figure 1:**
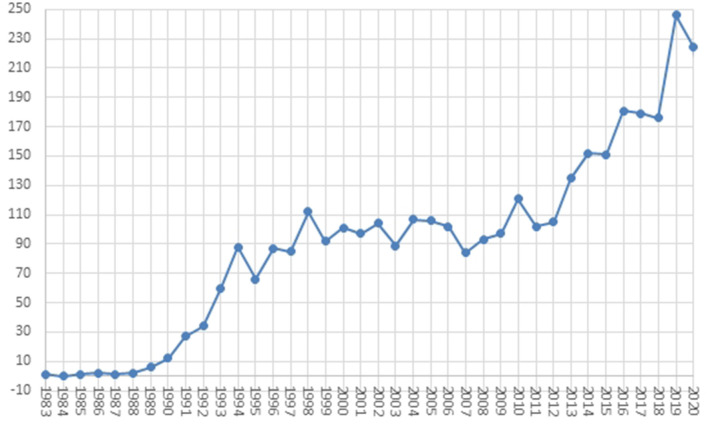
Trend in the number of articles.

Reference publication year spectrography analysis has shown (Figure 1) that the knowledge development in lipoprotein research goes a long way back to the Heberden (18) paper on breast disorders published in 1,772. The following influential paper published by Anitchkov and Chalatov in 1913 (19) introduced the so-called lipid hypothesis of atherosclerosis (20). More intensive research began in the 1960 and 1970s with Bergs (21) paper introducing the lipoprotein biology in humans and Friedwald and Fredrickson (22) paper introducing a new method to estimate the LDL-C concentration without ultracentrifuge. More recent influential publications are concerned with research on human apo(a) (23), assessing the risk of myocardial infarction using Lp(a) level (24), the implication of clinical trials for cholesterol education programs (25) and the relation of Lp(a) concentration on the risk of various forms of CVD (26–28).

**Figure 2:**
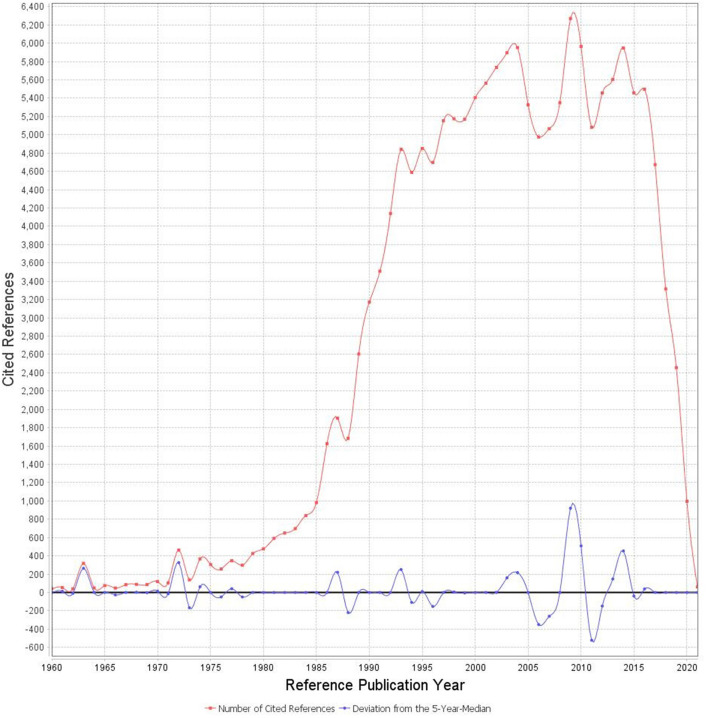
The spectrogram of the lipoprotein(a) research literature production.

The dynamics of the research literature production is shown in Figure 2. The first papers describing Lp(a) in the period indexed by Scopus were published in 1983 (29, 30). After that, until 1986, literature production was sparse (maximal two publications per year). In 1987, the first rapid development phase began with the recognition of homology between cDNA sequence of Lp(a) and plasminogen, while the interest in Lp(a) research declined by 1994 with two prospective trials, yielding no association of Lp(a) with CVD. The next phase, lasting till 2013, was characterized by slow linear growth in productivity, followed by the second rapid development phase. The maximal productivity was reached in 2019 with 246 publications. The distribution of paper types shows that Lp(a) research has started to establish its base of core papers; however, the exponential trend in publications and a low number of influential papers shows that research is well past its infancy stage. On the other hand, it is yet to reach a stable maturity, usually indicated by the stabilization of the number of new papers being published; therefore, some basic concepts in the research area have yet to be established.

### Geographical Research Dispersion and Country Co-operation

Bibliometric country and institution analysis may reveal influential research teams and potential collaborators and help researchers establish international or national Co-operation (31). The analysis showed that research production was distributed among 106 countries from all continents (Table 1). The most productive country by far was the United States of America (USA) (*n* = 1,134) with almost one-third of the whole literature production, followed by Germany (*n* = 335) and the United Kingdom (*n* = 319), both contributing ~10% to the overall literature production. The research literature productivity of the 10 most productive countries in the last 5 years was comparable to the overall productivity, with few exceptions: Italy dropped to the seventh rank, Austria to 11th and France to the 12th, China became the 6th, and Greece the 9th.

**Table 1:** Ten most productive countries.

**Country**	**Total number**	**Number of publications**
	**of publications**	**in last 5 years**
United States	1,134	313
Germany	335	116
United Kingdom	319	101
Italy	278	67
Canada	197	88
Australia	184	79
Spain	179	44
Netherlands	175	67
France	162	39
Austria	126	41

The top 10 countries produced more than 87% of all research literature. The top 10 countries are the leading industrial countries with well-developed economies and well-funded health systems. From the G7 countries, only Japan is outside the top 10 most productive 10 countries; however, it is placed immediately after and is the 11th.

The four most productive institutions were located in the USA and are the Harvard Medical School (*n* = 117), University of California, San Diego, USA (*n* = 103), Brigham and Women's Hospital, USA (*n* = 97), and University of Washington, USA (*n* = 75). University of Western Australia (*n* = 74) ranked fifth as the most productive Non-USA institution, followed by UMC of the University of Amsterdam, Netherlands (*n* = 70), Inserm, France (*n* = 69) and Royal Perth Hospital, Australia (*n* = 62). In the last 5 years, the productivity of Australian institutions increased, namely University of Western Australia (*n* = 44) become the most and Royal Perth Hospital (*n* = 40) the third most productive institutions and are together with the Harward Medical School, University of California and also other more productive institutions viable candidates for collaboration.

The above results indicate a regional concentration of research and knowledge to economically strong and most developed countries with well-advanced health and research systems. Such centralization is undesirable not only from a scientific point of view but also for evidence-based policy-making. To improve the overall state of Lp(a) research, researchers in low- and middle-income countries must develop collaborations with high-income countries. Collaborative research not only contributes to a more comprehensive Lp(a) understanding but might contribute to building a shared base of knowledge, data, innovations, evidence and Lp(a) research paradigms.

**Figure 3:**
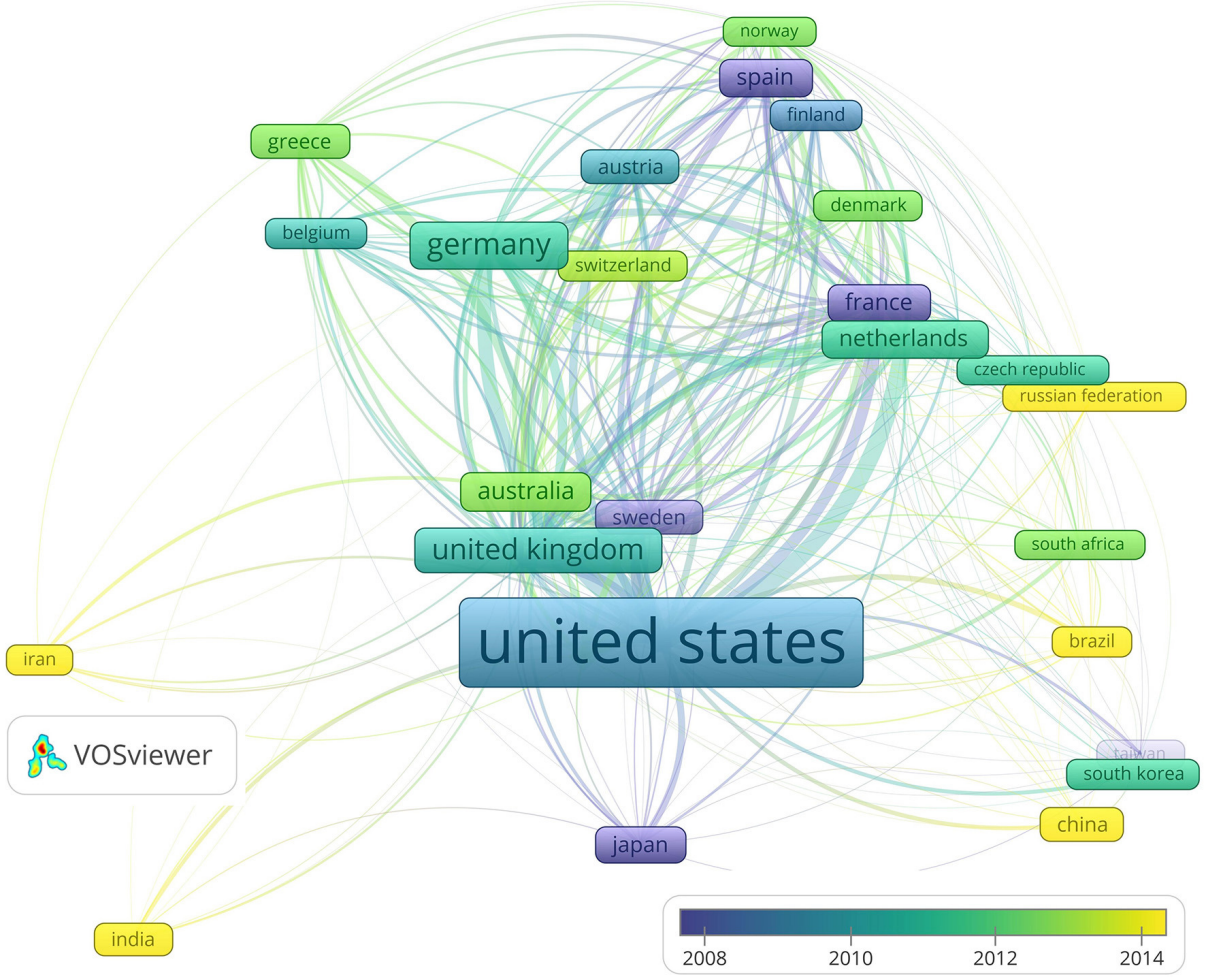
Country Co-operation network based on Co-authorships between 30 most productive countries.

In addition to the spatial bibliometrics, the analysis of the already established Co-operations links could disclose existing successful collaborations and indicate opportunities to strengthen current Co-operations and launch new ones. The country Co-operation network based on Co-authorship is shown in Figure 3: it indicates that the USA, UK, France, Canada, Netherlands, China, UK, and Germany established most international links. The USA Co-operates with 52 countries, the UK with 43, France with 40, Canada with 38, the Netherlands with 39, Germany with 38, and China with 37. The strongest existing links are between the USA and Canada, USA and UK and USA and France; Germany, France and Spain have strong links with other EU countries and Australia. From the most productive Asian countries, China, Japan, and South Korea have most links, however not among themselves. On average, the Co-operation inside single continents is weaker than inter-continental Co-operation, except for the links mentioned above.

Figure 3 also reveals that, on average, France, Spain, Sweden, and Japan publications are according to the average publishing years, the oldest and Iran, India, Russian Federation, Brazil and Chine the youngest. Post-analysis shows that the most productive country, namely the USA has a long–term linear trend in publications number growth, while countries from the second group like China, India and the Russian federation have a short but exponential growth.

Research funding is critical in performing successful research and amplifying its impact. Bibliometric analysis of publications funding recognizes the most prolific funding bodies and thus supports researchers in obtaining funding (32). The 12 most prolific institutions funding Lp(a) research are shown in Table 2. Half of the institutions are governmental, and the other half are pharmaceutical firms. It is interesting to note that they are mainly located in the USA.

**Table 2:** Most prolific funding institutions.

**Funding institution**	**Number of funded publications**
National Heart, Lung, and Blood Institute, United States	289
National Institutes of Health, United States	243
U.S. Department of Health and Human Services	116
Amgen, United States	87
Sanofi, France	74
Pfizer, United States	73
National Center for Research Resources	67
National Institute of Diabetes and Digestive and Kidney Diseases	53
AstraZeneca, United Kingdom	50
Merck	49
Novartis	49
American Heart Association, United States	40

### Prolific Source Titles

The most prolific journals are presented in Table 3. The majority of the Lp(a) research is published in journals related to Atherosclerosis or Lipidology. All top 10 journals have high CiteScore journal impact factors (CiteScore 2020 counts the citations received in 2017–2020 to articles, reviews, conference papers, book chapters and data papers published in 2017–2020, and divides this by the number of publications published in 2017–2020). They are ranked in the highest quartile and three journals even in the top five per cent of cardiology research journals. The benefit of the source title analysis is that it could identify the first best sources where researchers can find relevant information about Lp (a) research and second journals where prospective authors might publish their studies (33). The high impact factors of most prolific journals reveal that Lp(a) research is becoming an essential and influential part of cardiology research.

**Table 3:** Ten most prolific journals in lipoprotein(a) research.

**Journal**	**CiteScore 2020**	**Total number of publications**	**Number of publications 2017–21**
Atherosclerosis	6.7	143	43
Journal of Clinical Lipidology	7.2	69	42
Current Opinion in Lipidology	7.0	61	17
Journal of the American College Of Cardiology	33.1	60	29
Arteriosclerosis Thrombosis and Vascular Biology	12.5	56	15
European Heart Journal	20.4	54	30
Circulation	31.5	50	8
Journal of Lipid Research	8.9	49	17
Atherosclerosis Supplements	4.8	48	19
Current Atherosclerosis Reports	5.6	41	19

### Prolific Research Themes

We performed the content analysis on two levels, first on the high level based on the subject categories denoted by Scopus and second on the meso-level based on authors keywords Co-occurrences. The most prolific subject categories were Medicine (*n* = 3,106), Biochemistry, Genetics and Molecular Biology (*n* = 920), Nursing (*n* = 327), Pharmacology, Toxicology and Pharmaceutics (*n* = 225, Immunology and Microbiology (*n* = 56), and Agricultural and Biological Sciences (*n* = 54).

**Figure 4:**
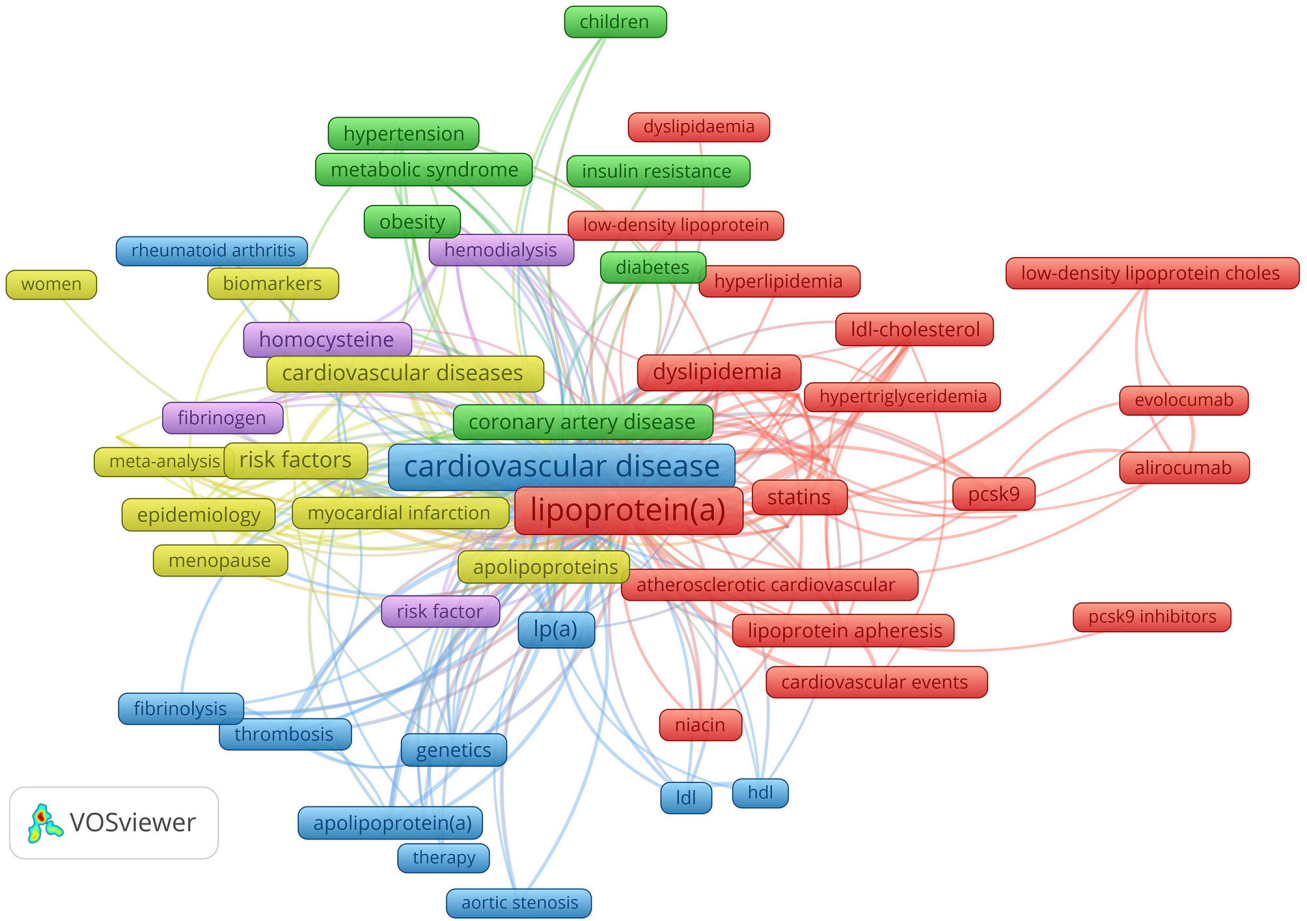
Author keywords (with 20 or more occurrences) cluster landscape. Author keywords cluster landscape revealed five research themes: Lp(a) and inflammation (violet), Lp(a) cardiovascular diseases (blue), Lp(a) in women (yellow), prevention and treatment of coronary artery disease in diabetic patients (green), lipid abnormalities and treatment (red).

Synthetic knowledge synthesis performed on author keywords cluster landscape (Figure 4) resulted in five themes and 12 categories presented in Table 4.

**Table 4:** Results of the synthetic knowledge synthesis.

**Theme**	**Representative author keywords**	**Categories**
Lp(a) and inflammation (violet)	Lipoprotein (a) (488), fibrinogen (47), c-reactive protein (55), homocysteine (75)	Lipoprotein (a), fibrinogen, C-reactive protein and homocysteine as risk factors in CVD
Lp(a) in Cardiovascular Diseases (blue):	Cardiovascular disease (458); atherosclerosis (351); thrombosis (36); inflammation (106); Lp(a) (134); apolipoprotein (64); fibrinolysis (37); LDL (45); HDL (27)	Atherosclerosis, thrombosis, inflammation; fibrinolysis and lp(a); cardiovascular diseases and apolipoprotein(a); atherosclerosis, lp(a). LDL and HDL
Lp(a) in women (yellow)	Woman (21); hormone replacement therapy (28); menopause (40; cardiovascular diseases (103); lipids (195), lipoproteins (199); risk factor (131); coronary heart disease (92); stroke (40); myocardial infraction (48).; epidemiology (61); meta-analysis (20)	Hormone replacement therapy during menopause in relation to cardiovascular diseases and lipids; lipoprotein as a risk factor in coronary diseases; epidemiology and meta-analysis studies on stroke and myocardial infraction
Prevention and treatment of coronary artery disease in diabetic patients (green)	Cardiovascular risk factors (54); hypertension (84); obesity (60); metabolic syndrome (53); coronary heart diseases (87); diabetes (89); prevention (51); children (41); treatment (20)	Cardiovascular risk factors (hypertension, obesity, metabolic syndrome, lipoprotein (a)) and coronary artery diseases in diabetes patients; obesity as a cardiovascular risk in children:
Lipid abnormalities and treatment (red):	Lipoprotein(a) (566), fibrinogen (47), c-reactive protein (55), homocysteine (75), dyslipidaemia (138), cholesterol (156), hypercholesterolemia (71), familial hypercholesterolemia (87), triglycerides (73), pcsk9 (78), apheresis (83), alirocumab (30), ezetimibe (20), statin (97), evolocumab (27), niacin (39), cardiovascular events (37)	Lipid abnormalities and drugs (statin, ezetimibe, alirocumab); lipid abnormalities; lipoprotein apheresis and cardiovascular events;

*The numbers in parentheses represent the number of papers in which the author keyword occurred*.

#### Lp(a) and Inflammation (Violet)

The central role of inflammation and oxidative stress in the process of atherosclerosis has been confirmed in several studies (19–22, 34). Similarly to the well-known atherogenic potential of LDL-C, Lp(a) is also prone to oxidative modifications and the formation of pro-inflammatory and pro-atherogenic particles that perpetuate the progression of atherosclerotic plaques. Lp(a) as an LDL-like particle, directly infiltrates the vascular wall, attracts inflammatory cells and promotes atherogenesis. Furthermore, oxidized phospholipids, frequently bound to the surface of Lp(a), activate endothelial cells and thereby facilitate an increased transendothelial migration of monocytes and promote their vascular wall infiltration with subsequent inflammatory cytokine release (35, 36). Due to its similarity with plasminogen, Lp(a) might also exert an inhibitory effect on fibrinolysis and thereby promote thrombogenesis (37).

Despite the abundant evidence of pro-inflammatory actions of Lp(a) on macrophages and vascular endothelium, the link between the level of Lp(a) and high-sensitivity C-reactive protein (CRP) concentration has not been consistently demonstrated (38). However, the effect of inflammation on the Lp(a)-associated cardiovascular risk has also been investigated, suggesting the potential benefit of Lp(a)- lowering only in the subgroup of optimally treated high-risk patients with elevated CRP >2 mg/L (39, 40). The relation of Lp(a) and other pro-inflammatory molecules like homocysteine has also been studied. Simultaneous elevation of homocysteine and Lp(a) was found to increase the risk of CAD in females, suggesting an interactive effect of homocysteine and Lp(a) on the risk of CAD (41).

#### Lp(a) in Cardiovascular Diseases (Blue)

There is growing evidence that Lp(a) is associated with ASCVD (42–45). The more extensive epidemiologic evidence of the association between Lp(a) and ASCVD arises from the Copenhagen City Heart Study general population data (2008), showing a stepwise increase in the risk of myocardial infarction with increasing Lp(a) levels (42). In 2009, the Emerging Risk Factors Collaboration published a meta-analysis of 39 long-term prospective studies and confirmed an independent link between Lp(a) and ASCVD (26). In Mendelian genetic studies, lower kringle IV-type 2 repeat numbers in the apolipoprotein(a) gene and subsequent higher Lp(a) concentrations were associated with myocardial infarction, peripheral arterial disease, stroke and CAVS (43–45). Lp(a) levels >50 mg/dl have been demonstrated in 20% of the general population, being highly overrepresented in patients with CVD (5).

While the link between Lp(a) and cardiovascular events has been consistently demonstrated in general population studies, more extensive evidence about the prognostic value of Lp(a) in secondary prevention patients has been introduced recently. In a recent Danish population-based study, Lp(a) predicted future cardiovascular events in secondary prevention patients, hypothesizing a 20% reduction of cardiovascular events by lowering Lp(a) by 50 mg/dl (46). Similarly, a recent meta-analysis found Lp(a) a risk predictor for future cardiovascular events in patients with an established coronary artery disease (47). In 2020, the most abundant evidence to date, including 460 506 middle-aged primary and secondary prevention United Kingdom Biobank participants with 11.2 years of median follow-up, confirmed a linear relationship between Lp(a) and ASCVD across the Lp(a) distribution (48). Although Lp(a) concentrations differed in white, South Asian, black and Chinese individuals, a similar relationship between Lp(a) level and ASCVD risk was maintained in all racial/ethnic subgroups (48). In the latter study, a high Lp(a) concentration defined as ≥150 nmol/L was present in 12.2% of participants without and 20.3% of those with the preexisting ASCVD and was associated with hazard ratios for cardiovascular events of 1.50 (95% CI, 1.44–1.56) and 1.16 (95% CI, 1.05–1.27), respectively (48).

The evidence associating Lp(a) with the incidence and progression of peripheral arterial disease is also growing. The large European Prospective Investigation of Cancer (EPIC)-Norfolk prospective population study with 1.3 million person-years demonstrated a 37% increased risk of peripheral arterial disease for 2.7-fold higher Lp(a) concentration (49). Most other prospective studies yielded similar results, while only a few of them did not confirm this association (50).

The predictive role of Lp(a) for stroke is more controversial and less well-established than for ischemic heart disease. In the EPIC-Norfolk study, Lp(a) level was not associated with ischemic stroke, and some other studies were inconclusive (49, 51). The most recent meta-analysis of 41 studies demonstrated an association of Lp(a) with ischemic stroke and predominantly the large artery atherosclerosis-associated subtype of ischemic stroke (52). Interestingly, Lp(a) was also linked with intracerebral hemorrhage (52).

In recent years, the evidence revealing the association of Lp(a) with CAVS has accumulated (53–55). A transcriptomic analysis identified genes potentially influenced by Lp(a), which were involved in cardiac aging, chondrocyte development and inflammation (54). Lp(a) was shown to upregulate genes involved in osteogenic differentiation and promote aortic valve calcium deposition *In vitro* (54, 56–58). In a human multimodality imaging study, patients in the top Lp(a) tertile with Lp(a) >35 mg/dl showed an increased aortic valve ^18^F-sodium fluoride (^18^F-NaF) positron emission tomography (PET) valvular calcification activity, much faster progression of valvular computed tomography calcium score, accelerated hemodynamic deterioration on echocardiography and increased risk for aortic valve replacement and death (58). Similarly, in the Aortic Stenosis Progression Observation: Measuring Effects of Rosuvastatin (ASTRONOMER) trial, patients with the highest Lp(a) had an increased rate of disease progression and increased need for aortic valve interventions (55).

Using machine learning, Missala et al. (59) found a link between systemic autoimmune diseases (rheumatoid arthritis, systemic lupus erythematosus) and increased Lp(a) as well as its association with increased atherosclerotic risk in these patients. Oxidized Lp(a) is supposed to trigger anti-Lp(a) antibodies in systemic autoimmune disorders, promoting inflammation and accelerating atherogenesis (59). The clinical significance of anti-Lp(a) antibodies and other mechanisms connecting autoimmune disorders and accelerated atherosclerosis are still poorly recognized and warrant further research. Another application of machine learning revealed that non-diabetic patients with chronic kidney disease have a hidden Pro-atherogenic lipoprotein profile, comprising an increased number of very-low- density lipoprotein (VLDL) particles and reduced LDL-particle size (60).

#### Lp(a) in Women (Yellow)

Women, on average, develop CVD ~7–10 years later than men; however, the menopause transition is associated with an atherogenic lipid profile, including increasing Lp(a) concentration and equalizing the CVD risk profile with men (61, 62). Furthermore, the Lp(a) >30 mg/dl was identified as an independent risk factor of coronary artery disease (CAD) in Post-menopausal women (62). Recent studies suggest that hormone replacement in women might reduce CVD risks and is cardioprotective, but most authors do not recommend lowering Lp(a) due to potential adverse effects (breast cancer, stroke, thrombosis), outweighing its benefits (63, 64).

#### Prevention and Treatment of Coronary Artery Disease in Diabetic Patients (Green)

Dyslipidemia is a frequent finding in patients with obesity, type 2 diabetes mellitus and metabolic syndrome. In these patients, insulin resistance is common. The characteristic pattern of atherogenic dyslipidemia with elevated triglycerides, small and dense LDL-particles, and low high-density cholesterol is frequently encountered (65). Weight reduction, diet modification and physical activity have demonstrated a beneficial effect in dyslipidemia treatment. However, Lp(a) has an autosomal co-dominant inheritance pattern with only a modest impact of lifestyle interventions.

Remarkably, a meta-analysis of prospective cohort studies has demonstrated an inverse association between Lp(a) levels and the risk of type 2 diabetes (66). The risk at Lp(a) concentrations below 7 mg/dl was more pronounced (66). In a cross-sectional analysis of Lp(a) concentrations from 36 studies, diabetic patients had 11% lower Lp(a) levels (95% CI: 4–17) compared with non-diabetic controls (26), while the reason for this inverse association remains controversial. Although some prior studies failed to show the link between Lp(a) and ASCVD in diabetic subpopulations, recent data confirms Lp(a) as a risk factor for ASCVD also in diabetic patients (67). In a recent meta-analysis including patients with type 2 diabetes mellitus, a higher Lp(a) level was associated with an increased incidence of diabetic nephropathy (68).

#### Lipid Abnormalities and Treatment (Red)

The primary goal of dyslipidemia treatment is LDL-C reduction using statins, and LDL-C target levels are specified based on the CVD risk profile (69–72). The LDL-C reduction primarily drives the beneficial effect of statins on cardiovascular outcomes, but evidence suggests additional “pleiotropic” anti-atherosclerotic activities, such as modulation of atherosclerotic vascular inflammation (73). A 9–24% increase in Lp(a) following statin treatment has been demonstrated in recent studies (74). This increase might contribute to the high residual CVD risk in statin-treated patients (71).

When using Friedewald equation, an adjustment for Lp(a)-cholesterol contribution to LDL-C is recommended (30% of total Lp(a) mass), especially in patients with extremely high Lp(a) levels (>1,000 mg/L) (75). In patients with recurrent events despite attaining LDL-C goals, high residual CVD risk might be associated with significantly increased Lp(a)-cholesterol levels, accounting for most of their LDL-C result. Adjusting LDL-C for Lp(a) contribution is also crucial in patients with familial hypercholesterolemia (FH), since they frequently have high Lp(a) levels attributed to impaired catabolism by dysfunctional or reduced hepatic LDL-receptors. Significantly increased Lp(a) leads to misinterpretation of an LDL-C result and thereby lowers diagnostic accuracy of the common FH diagnostic criteria (Dutch Lipid Clinic Network, Simon Broome criteria) used for phenotypic FH diagnosis (75). In the Spanish Familial Hypercholesterolemia Cohort Study (SAFEHEART), elevated Lp(a) >500 mg/L was recognized as an additional independent CVD risk factor in already high-risk patients with FH (76).

Since effective treatment of increased Lp(a) lacked until recently, the growing publication interest regarding Lp(a) in the last years might reflect the recently introduced promising treatment options. Proprotein convertase subtilisin/kexin type 9 (PCSK9) inhibitors (evolocumab, alirocumab) are widely-used and effective monoclonal antibodies targeting PCSK9, primarily used in combination with statins and ezetimibe to lower LDL-C concentration in very high-risk patients, yielding a modest—up to 30% reduction of Lp(a) level (77). Inclisiran, a novel small interfering ribonucleic acid (RNA) drug, demonstrated 18.6–25.6% Lp(a) reduction by inhibiting hepatic PCSK9 synthesis (78). While the reduction of major CVD events with PCSK9 inhibitors has been confirmed in two large independent trials, their clinical benefit has been attributed mainly to the reduction of LDL-C, and the clinical impact of the Lp(a) reduction has not been evaluated yet. However, a recent meta-analysis showed a consistent and comparable reduction of Lp(a) and Non-high-density cholesterol (Non-HDL-C), apolipoprotein B, and triglycerides with an increase in HDL-C across all studied racial/ethnic groups, treated with a PCSK9 inhibitor (79). Niacin, mipomersen, and lomitapide also significantly decrease Lp(a) level, but unfortunately, adverse side effects limit their widespread use. Newer Lp(a)-lowering drugs, such as an antisense oligonucleotide for apo(a) (pelacarsen) and two small interfering RNA molecules targeting apo(a) messenger RNA, are currently being investigated with serum Lp(a) reductions of up to 90% reported, and clinical outcome studies are still anticipated (2). Due to the lack of treatment outcome studies, the Lp(a) treatment cutoffs and treatment goals have not been widely accepted yet.

Lipoprotein(a) apheresis has been considered in the HEART UK Lipoprotein apheresis guidelines for high-risk patients with progressive coronary artery disease with Lp(a) >60 mg/dl and LDL-C >125 mg/dl despite maximal lipid-lowering therapy (80). The clinical benefit of Lp(a) apheresis on CVD outcomes is currently tested in clinical trials (NCT02791802). However, the method is limited by high costs and limited availability (81).

### Research Gaps and Future Directions of Lp(a) Research

There are many knowledge gaps to be addressed in the future regarding Lp(a) genetics, production, metabolism and clearance.

Lipoprotein(a) level heterogeneity is mainly attributed to the kringle IV-type 2 copy number in the Lp(a) gene locus (*LPA*). However, numerous single nucleotide polymorphisms in the *LPA* gene are emerging as potential Lp(a) level modifiers (82). Recent association studies have also demonstrated the contribution of other Lp(a) regulating genes outside the *LPA* locus (83). However, the impact of other genes on Lp(a) homeostasis needs further research.

Lipoprotein(a) levels have been considered relatively stable over a lifetime unless affected by clinical conditions like liver or kidney diseases (77). In line with this hypothesis, current European guidelines for managing dyslipidaemias recommended measuring Lp(a) level ones in every adult person (6). Recently, modest temporal variability in Lp(a) level of up to 20% was demonstrated, challenging previous recommendations and suggesting a mean of 2 Lp(a) measurements be obtained at different occasions for more precise risk assessment (84). Most guidelines for managing dyslipidaemias agree on using Lp(a) as a potential CVD risk modifier, especially in moderate-risk patients (6). However, due to the lack of universally accepted Lp(a) cutoffs, uncertainty exists about incorporating Lp(a) into ASCVD risk models. The distribution of reported Lp(a) levels in the general population varies more than 200-times, being considerably influenced by racial and ethnic factors, with the highest levels in those of African descent (84). However, more research is needed to better understand the impact of race and ethnicity on Lp(a) level, to estimate the risks of elevated Lp(a) in different patient populations, including those with FH, and to define subgroups of patients with largest benefit from Lp(a)-lowering medications.

The understanding of Lp(a) assembly from apo(a) and apoB is still under-recognized, and the routes of excretion are not entirely defined. The most extensive data showed apo(a) isoform size-dependent secretion. Large apo(a) isoforms are increasingly degraded by proteasomes and are associated with lower Lp(a) concentration (85). The Lp(a) clearance mechanisms are being investigated, and the hepatic and potential extrahepatic receptors await identification. The impact of LDL-receptor mediated catabolism of Lp(a) in FH patients as well as the influence of LDL-lowering medications on Lp(a) catabolism are a matter of ongoing research.

Uncertainty exists about the potential role of apo(a) isoform size in ASCVD and CAVS progression (86). Whether smaller apo(a) isoform sizes are associated with an increased risk independently of Lp(a) levels remains controversial and needs further clarification.

Another major issue regarding Lp(a) is the lack of global standardization of Lp(a) assays. Furthermore, Lp(a) sample handling and storing conditions might also affect Lp(a) result. Immunoassays using specific apo(a) antibodies may result in under-or overestimation of Lp(a) levels due to the variable kringle IV-type 2 repeats in the apo(a) structure (87). While Lp(a) levels have long been expressed in different units worldwide with unresolved conversion issues and uncomparable results, most current recommendations suggest using molar concentration, reflecting the number of Lp(a) particles in nanomoles per liter rather than the Lp(a) mass concentration affected by the isoform size (6). Moreover, while pro-inflammatory and pro-calcific effects of Lp(a) have been extensively studied, there are still unanswered questions regarding the impact of Lp(a)-mediated inhibition of fibrinolysis in atherothrombotic events (88). Despite the extensive homology between apo(a) and plasminogen, there is still ongoing controversy about the potential link between increased Lp(a) levels and venous thromboembolism (89).

Finally, a placebo-controlled outcome trial using an antisense oligonucleotide pelacarsen is currently in progress, as the expected clinical benefits of pharmacological Lp(a) lowering still need confirmation (Lp(a)HORIZON trial). In the future, cascade screening for extremely high Lp(a) in patients with and without FH might help early recognize affected individuals and enable early treatment with largest expected benefit.

### Hot Topics of the Recent Lp(a) Research

The hot-topics analysis revealed that the Lp(a) related research in the last 4 years had been focused on:

- Apolipoprotein B/A_1_ ratio vs. Lp(a) as potential CVD predictors,
- Lp(a) and aortic stenosis,
- Lp(a) and inflammation,
- Lp(a) and triglycerides, and
- Lp(a) and anti-PCSK9 directed monoclonal antibodies.

While the link between Lp(a) and ASCVD has been robustly studied and confirmed in many previous studies, CAVS has been a hot topic of more recent Lp(a) research. In the large EPIC-Norfolk Prospective Population Study with a follow-up of more than 19 years, the ratio between atherogenic apoB and antiatherogenic apoA1-containing particles (apoB/A1 ratio) was associated with the incidence of CAVS, especially in young and female participants. In the same study, Lp(a) was even more predictive than the apo B/A1 ratio for CAVS independently of age, gender, LDL-C, and concomitant CAD (90).

Most previous studies with statins did not demonstrate a beneficial effect of LDL-C- lowering on the progression of CAVS (55), while the effect of Lp(a) reduction has not been studied yet. Interestingly, high levels of PCSK9 protein, associated with higher atherogenic lipoprotein levels including Lp(a), correlated with CAVS and promoted aortic bioprosthesis calcification (91, 92). Moreover, in a recent meta-analysis of 10 genetic association studies, PCSK9 R46L-loss-of-function mutation was linked with a lower risk of CAVS (93). Recent experimental data based on the PCSK9 knockout mouse model suggest a direct detrimental role of PCSK9 in valvular interstitial cell calcification (94). In a recently published exploratory analysis of the Further Cardiovascular Outcomes Research With PCSK9 Inhibition in Subjects With Elevated Risk (FOURIER) trial, a PCSK9 inhibitor evolocumab decreased the incidence and progression of CAVS and the need for aortic valve replacement in patients with CVD (95). The hypothetic beneficial effect of PCSK9 inhibitors is further being tested in one registered clinical trial (NCT03051360), comparing aortic calcium score progression in patients treated with monoclonal antibodies directed against PCSK9 and controls. To our knowledge, there is currently no study investigating the potential benefits of the PCSK9-lowering drug Inclisiran on the natural course of CAVS.

The hypothetical impact of the emerging Lp(a)-lowering drugs and the PCSK9-targeted therapy on the incidence and progression of CAVS will probably be a focus of aortic valvular research in the near future. The issue is essential since no other pharmacological agent has been proven effective in treating CAVS yet (95).

### Strength and Limitations

The main strength of our study is the use of a novel knowledge synthesis method and state-of-the-art bibliometric analysis and mapping tools. In this manner, we presented a comprehensive review of the Lp(a) research and revealed several essential characteristics of the Lp(a) research literature production. We believe that this study is the first to analyse the lp(a) research and knowledge development on both the knowledge and meta-knowledge levels, thus revealing new evidence about the Lp(a) research and possible future directions, thus enabling researchers to focus their efforts. We introduced the map of the lp(a) research themes and associations between them on the knowledge level. We also identified hot topics and future trends. On the meta-knowledge level, we determined the dimensions of Lp(a) research processes like most productive countries and institutions, Co-operation patterns between countries, most prolific source titles, and the knowledge development timeline, allowing researchers to determine appropriate partners and knowledge resources when starting research.

However, this research study did have some limitations. First, it should be noted that we used the Scopus database for this study; therefore, if other bibliographic databases were used, the results of this study might be somewhat different, or some important papers could be left out of the historical analysis. However, the Scopus database is the largest bibliographic database covering almost 41,000 source titles, and we believe that selecting other databases would not significantly impact the outcomes of the study. The search was defined in the manner to harvest only the papers that contained lipoprotein(a) in the title, abstract or keywords. Papers presenting general lipoprotein research were not included in the analysis, consequently some historical roots might be missed. Furthermore, the thematic part of the analysis was qualitative and, consequently, with a higher risk of subjective conclusions.

## Concluding Thoughts

Our study represents the first holistic and synthetic knowledge synthesis of Lp(a) research to the best of our knowledge. It showed that Lp(a) had been recognized as an essential risk factor for ASCVD and CAVS. Our bibliometric analysis of Lp(a) in CVD demonstrated increasing interest in Lp(a) research in the recent years, potentially influenced by recently introduced treatment options with PCSK9 inhibitors and especially highly effective emerging Lp(a)-reducing drugs like antisense oligonucleotides and RNA interference agents.

The analysis of author keywords revealed that Lp(a) was most intensively studied regarding inflammation, atherosclerosis, CVD risk, treatment options, and hormonal changes in post-menopausal women. Our hot topic analysis revealed new perspectives of potential lipoprotein research. Further studies are needed to clarify the impact of Lp(a) reduction with novel drugs, and the role of PCSK9 inhibition to potentially slow down the progression of CAVS.

The review can help researchers and practitioners to understand the broader aspects of Lp(a) research and its translation into clinical practice. Additionally, it can inform a health professional to develop a perspective on the most important research themes and serve as a starting point for further research.

## Author Contributions

DS: conceptualization, data curation, and writing—original draft. HB and JZ: data curation and writing—review & editing. PK: conceptualization, writing—original draft, data curation, formal analysis, validation, and writing—original draft. AS and VK: conceptualization and writing—original draft. FN, TZ, and MK: conceptualization, data curation, and writing—review & editing. All authors contributed to the article and approved the submitted version.

## Conflict of Interest

MK was employed by Semantika Research, Semantika d.o.o. The remaining authors declare that the research was conducted in the absence of any commercial or financial relationships that could be construed as a potential conflict of interest.

## Publisher's Note

All claims expressed in this article are solely those of the authors and do not necessarily represent those of their affiliated organizations, or those of the publisher, the editors and the reviewers. Any product that may be evaluated in this article, or claim that may be made by its manufacturer, is not guaranteed or endorsed by the publisher.
